# Structural Characterization and Expression Profiling of Ethylene Biosynthetic Genes During AgNO_3_-Induced Sex Reversal in Bitter Gourd

**DOI:** 10.3390/ijms27135980

**Published:** 2026-07-03

**Authors:** Da Zhang, Kanghua Du, Zhong Dan, Xiaomei Li, Lingfeng Bao, Guangping Chen, Jie Jin, Jixian Ma, Wanfu Mu

**Affiliations:** Tropical Eco-Agriculture Research Institute, Yunnan Academy of Agricultural Sciences, Yuanmou 651300, China

**Keywords:** *Momordica charantia*, ethylene biosynthesis, AgNO_3_-induced sex reversal, ACC synthase (ACS), 3D structural modeling, expression profiling

## Abstract

Ethylene biosynthetic enzymes, 1-aminocyclopropane-1-carboxylate (ACC) synthase (ACS) and ACC oxidase (ACO), participate in the floral sex differentiation of bitter gourd (*Momordica charantia*). However, the relationship between their structural features and developmental expression patterns remains to be further clarified. In this study, eight *McACS* and five *McACO* genes were identified using the Dali-11 reference genome. AlphaFold 3-based modeling showed structural differences between the two families, particularly regarding the diverse C-terminal flexibilities of McACS proteins. Targeted qRT-PCR profiling during the critical 1.0–3.0 mm early floral bud stage revealed that *McACS7*, a structurally stable Type III member, along with *McACS1*, *McACS12*, and *McACO2*, were significantly upregulated during natural female flower development. Furthermore, treatment with silver nitrate (AgNO_3_), an ethylene perception inhibitor, suppressed the transcription of these synthesis genes to basal levels and induced hermaphroditic flower formation. Instead of fully elucidating the downstream molecular mechanisms, these findings provide robust candidate-gene evidence and transcriptional profiling that link the ethylene biosynthetic machinery to the chemically induced sex reversal process, thereby laying a solid foundation for future functional characterization.

## 1. Introduction

Bitter gourd (*Momordica charantia* L.) is an economically significant crop cultivated across tropical and subtropical regions for its nutritional and medicinal value. As a typical monoecious species within the Cucurbitaceae family, its fruit yield is fundamentally determined by the ratio of female to male flowers [1]. Sex determination in cucurbits is a complex developmental process regulated by diverse endogenous and environmental factors, among which the phytohormone ethylene acts as the principal signal mediating feminization [2]. The molecular mechanisms of floral sex differentiation have been extensively studied in model Cucurbitaceae species, such as cucumber (*Cucumis sativus*) and melon (*Cucumis melo*). In these species, classical genetic models revealed that the F locus (*CsACS1G*/*CmACS7*) and the M locus (*CsACS2*/*CmACS7*) coordinately control female flower development, while the A locus (*CsACS11*/*CmACS11*) acts as a master regulator for carpel initiation [3,4]. These loci encode rate-limiting enzymes in the ethylene biosynthetic pathway, specifically 1-aminocyclopropane-1-carboxylate (ACC) synthase (ACS), which converts *S*-adenosyl-L-methionine to ACC, and ACC oxidase (ACO), which catalyzes the subsequent oxidation of ACC to ethylene [5,6]. Despite these elegant multi-gene models in model cucurbits, the specific orchestration of the ethylene biosynthetic machinery in bitter gourd (*M. charantia*), which possesses a highly distinct genomic landscape, remains incompletely characterized.

While ACO enzymes generally maintain a highly conserved structural fold to catalyze the final biosynthetic step, the ACS family exhibits extensive regulatory diversification [7]. In model plant species, ACS proteins are categorized into three main classes (Type I, Type II, and Type III) based on their C-terminal sequence motifs [8]. The extended C-terminal domains characteristic of Type I and II ACSs are intrinsically disordered regions. These extensions serve as primary target degrons for ubiquitin-mediated proteasomal degradation, resulting in rapid protein turnover [9]. In contrast, Type III ACSs lack this flexible extension, adopting a more compact three-dimensional conformation that has been reported to confer enhanced structural and proteolytic stability [9,10]. This structural dichotomy establishes C-terminal flexibility as a recognized molecular determinant of ACS half-life. By modulating the stability of specific ACS isoforms, plants precisely control the duration and amplitude of local ethylene production, a mechanism essential for directing complex developmental trajectories such as floral organogenesis.

In agricultural practice, hybrid seed production in bitter gourd is frequently hindered by the labor-intensive requirements of manual pollination and floral bagging. Deploying stable gynoecious lines as maternal parents significantly streamlines this process and reduces associated costs [1]. However, the absence of male organs in gynoecious lines constitutes a major obstacle to their routine maintenance and propagation. To overcome this reproductive barrier, silver nitrate (AgNO_3_), a potent competitive inhibitor of ethylene perception, is routinely applied to induce the development of male or hermaphroditic flowers yielding viable pollen [11,12]. Previous physiological studies suggest that ethylene may promote its own biosynthesis through an autocatalytic positive feedback loop during flower development [13,14]. Although the impact of AgNO_3_ on this regulatory mechanism has been investigated in model cucurbits, the specific transcriptional dynamics of key *ACS* and *ACO* genes during artificial sex reversal remain uncharacterized in *M. charantia*. Characterizing this process is critical to elucidate how external chemical stimuli perturb endogenous hormonal networks to dictate floral sex.

Recently, genetic mapping has localized gynoecious loci in bitter gourd, proposing genes such as *CTPS* as potential upstream regulators [15,16]. While these upstream genetic triggers are crucial, the consistent induction of male flowers following AgNO_3_ application demonstrates that local ethylene biosynthesis functions as the indispensable downstream effector executing carpel development [12]. To explore these downstream mechanisms, the *McACS* and *McACO* gene families were systematically identified utilizing the chromosome-level Dali-11 reference genome. By integrating AlphaFold 3-based three-dimensional structural modeling with time-series quantitative real-time PCR (qRT-PCR) profiling under natural and AgNO_3_-treated conditions, the structural determinants and transcriptional dynamics of these core enzymes were characterized. These findings establish a fundamental molecular basis for the downstream enzymatic machinery mediating feminization, highlighting specific *McACS* and *McACO* candidates for targeted functional validation and the molecular breeding of stable gynoecious lines.

## 2. Results

### 2.1. Identification of ACS and ACO Genes in M. charantia

Through a combined identification strategy utilizing HMM searches, BLASTP alignments, and CDD verification, 13 ethylene biosynthesis-related genes were identified in the *M. charantia* reference genome, comprising eight *McACS* and five *McACO* members (Table 1). Following standard nomenclature conventions, the *ACS* genes were designated as *McACS1*–*McACS5*, *McACS7*, *McACS10*, and *McACS12*, and the *ACO* genes as *McACO1*–*McACO5*, based on their phylogenetic relationships.

Physicochemical property analyses revealed distinct profiles for the two protein families (Table 1). For the McACS family, protein lengths ranged from 433 aa (McACS4) to 548 aa (McACS10), with corresponding molecular weights (MW) varying from 48.73 kDa to 60.17 kDa. Their isoelectric points (pI) exhibited a broad range, from 5.57 (McACS7) to 8.95 (McACS1). In stark contrast, the McACO family displayed a highly conserved physical profile, with protein lengths strictly restricted between 298 and 322 aa, and MWs ranging from 33.92 to 36.58 kDa. Furthermore, all McACO members presented acidic pI values (5.17–5.49). Subcellular localization predictions indicated that all 13 McACS and McACO proteins are localized in the cytoplasm, strictly consistent with their functional roles in cytosolic ethylene biosynthesis. The comprehensive properties of homologs in *Arabidopsis thaliana*, *C. sativus*, and *C. melo* are provided in Table S2.

### 2.2. Phylogenetic Analysis of the Ethylene Biosynthesis Gene Families

To evaluate the evolutionary relationships and orthologous lineages of the ethylene biosynthesis machinery, unrooted maximum likelihood (ML) phylogenetic trees were constructed using protein sequences from *M. charantia* and three reference species (*A. thaliana*, *C. sativus*, and *C. melo*).

**Figure 1:**
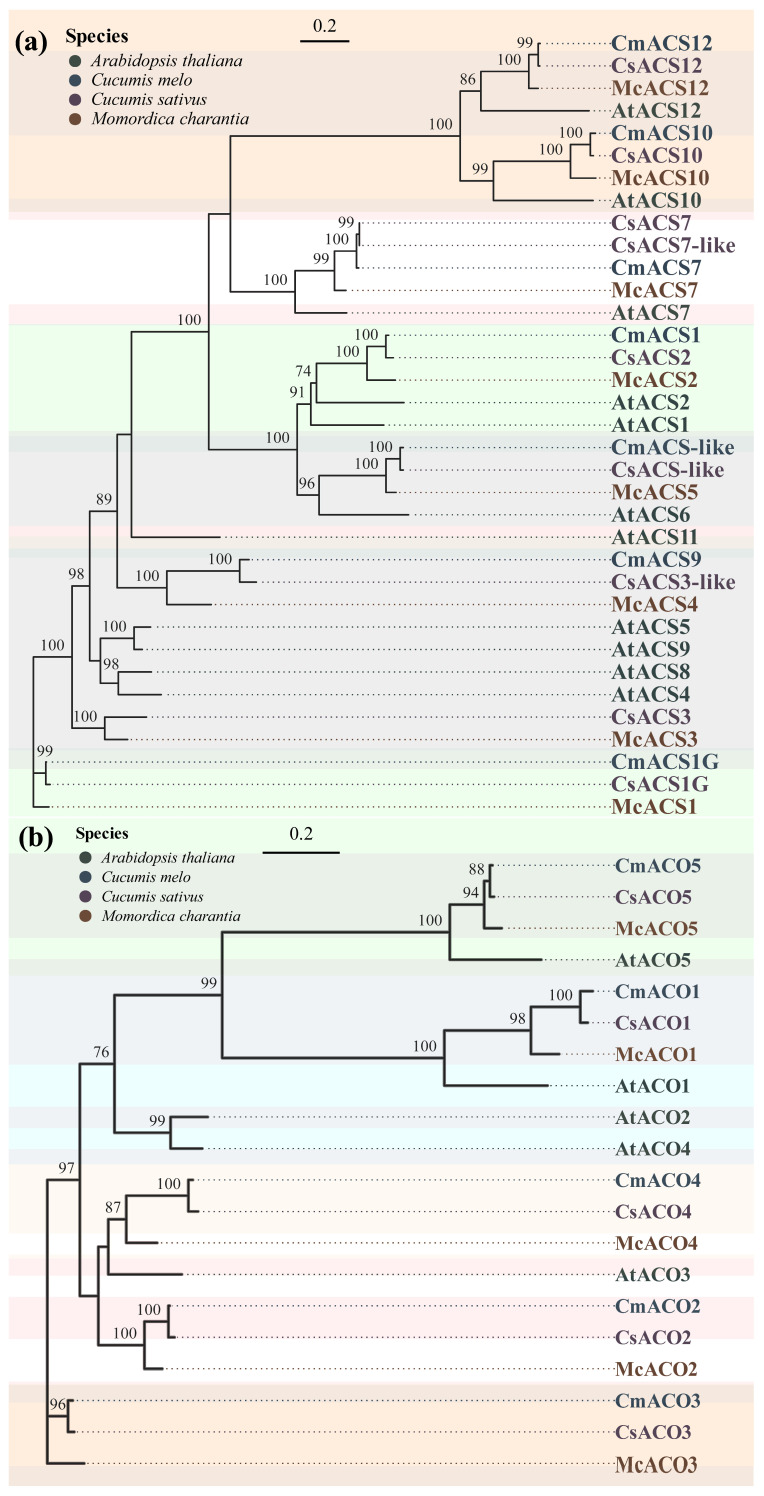
Phylogenetic analysis of the *ACS* and *ACO* gene families across multiple species. (**a**) Unrooted maximum likelihood (ML) tree of ACS proteins from *M. charantia* (Mc), *Arabidopsis thaliana* (At), *C. sativus* (Cs), and *C. melo* (Cm). The five distinct evolutionary clades (Type I, Type II, Type III, non-canonical, and basal) are highlighted by different background colors. (**b**) Unrooted ML tree of ACO proteins from the four representative species, demonstrating highly conserved lineage-specific orthologous groups.

The phylogenetic topology of the ACS family demonstrated that the eight McACS proteins are distributed across five distinct clades, consistent with the standard evolutionary categorization of plant ACS members (Figure 1a). Specifically, McACS2 and McACS5 clustered within the Type I clade; McACS3 and McACS4 resolved into the Type II clade; and McACS7 was identified as the sole member of the Type III clade. Additionally, McACS10 and McACS12 clustered with the non-canonical aminotransferase-like group, while McACS1 occupied a separate basal position. Notably, each McACS member clustered more intimately with its orthologs from the cucurbit species *C. sativus* and *C. melo* than with those from *A. thaliana*, indicating a highly conserved evolutionary lineage within the Cucurbitaceae.

In the ACO family, the five McACO proteins clustered into distinct orthologous groups with high bootstrap support, strictly pairing with their counterparts from cucumber and melon (Figure 1b). In contrast to the broad divergence observed in the ACS family, the *McACO* family exhibited a more condensed phylogenetic structure. The substantial branch lengths separating the major *ACS* clades, compared to the tight clustering of *ACO* members, suggest that while the *ACO* family remained evolutionarily constrained, the *ACS* subfamilies potentially underwent functional diversification following their ancient duplication.

### 2.3. Gene Structure and 3D Protein Folding Analysis

**Figure 2:**
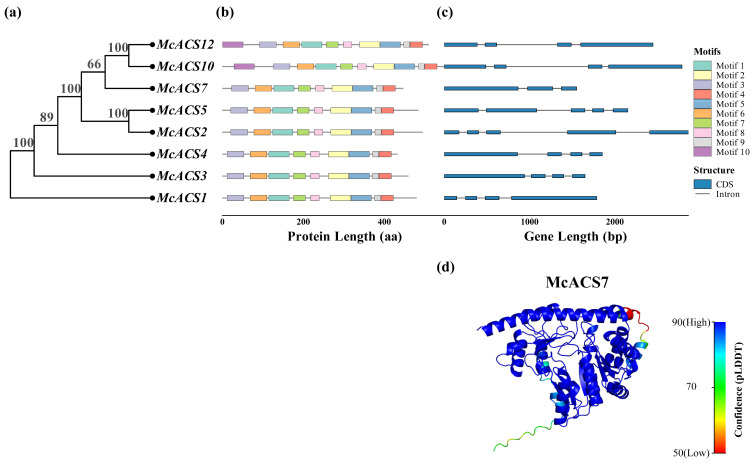
Phylogenetic, motif, and structural characterization of the *McACS* family. (**a**) Unrooted phylogenetic tree of the eight McACS proteins, classifying them into five distinct clades. (**b**) Conserved motif compositions. Distinct motifs are indicated by colored boxes. (**c**) Exon–intron architectures of the *McACS* genes. (**d**) AlphaFold 3-predicted three-dimensional structures of representative McACS7 proteins. The models are colored according to pLDDT confidence scores, highlighting the intrinsically disordered C-terminal extensions (red/orange) in contrast to the highly confident structural cores (blue).

To investigate the structural basis underlying the evolutionary divergence of the *McACS* family, conserved motifs and exon–intron organizations were mapped onto the established phylogenetic framework (Figure 2a). As delineated previously, the eight McACS proteins are distributed across five distinct clades. Motif analysis revealed a clade-specific distribution: the non-canonical members (McACS10 and McACS12) possessed all 10 predicted motifs, whereas the typical members (Type I, II, and III) lacked Motif 10 (Figure 2b). Furthermore, exon–intron organization varied within the family; most *McACS* genes contained four exons, while the Type III member *McACS7* contained only three, and the Type I members *McACS2* and *McACS5* contained five (Figure 2c). For a comprehensive interspecies comparison of motif distributions and exon–intron variations across cucurbit species, see Supplementary Figure S1.

To elucidate how these sequence-level variations translate into three-dimensional architectures, structural models were generated using AlphaFold 3 (Figure 2d). The core pyridoxal-5′-phosphate (PLP)-dependent transferase domains exhibited predominantly high predicted local distance difference test (pLDDT) scores (>90), indicating robust structural reliability across the family. However, substantial structural divergence emerged at the C-termini. Consistent with their phylogenetic clades, the basal, non-canonical, and Type I/II members exhibited extended, low-confidence (pLDDT < 50) C-terminal extensions appearing as disordered loops (Figure S2). In stark contrast, the sole Type III member, McACS7, completely lacked this flexible extension, adopting a highly compact and rigid C-terminal fold (Figure 2d). This three-dimensional structural dichotomy provides a structural framework consistent with the varying degrees of protein stability and turnover rates previously reported for specific ACS isoforms.

**Figure 3:**
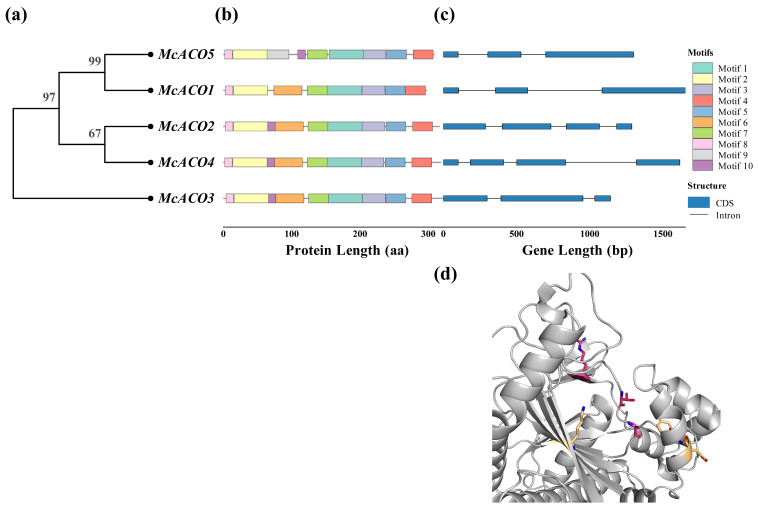
Phylogenetic, motif, and structural characterization of the *McACO* family. (**a**) Phylogenetic tree of the five McACO proteins. (**b**) Conserved motif compositions. (**c**) Exon–intron architectures of the *McACO* genes. (**d**) Magnified structural visualization of the McACO2 catalytic pocket, highlighting the precise spatial arrangement of the canonical His-Asp-His facial triad (His178, Asp180, and His235) required for Fe(II) coordination.

For the *McACO* family, a parallel structural analysis was performed. Aligned with their phylogenetic groupings (Figure 3a), motif composition varied slightly among the members: McACO2, McACO3, and McACO4 contained nine motifs (lacking Motif 9), McACO5 contained nine motifs (lacking Motif 6), and McACO1 retained eight motifs (lacking Motifs 9 and 10) (Figure 3b). Gene structures exhibited either three (*McACO1*, *McACO3*, and *McACO5*) or four (*McACO2* and *McACO4*) exons (Figure 3c). Notably, interspecies comparison revealed that while *McACO4* maintains four exons in bitter gourd, its orthologs in *Cucumis* species contain only two elongated exons, indicating lineage-specific structural variations (Figure S3).

To elucidate how these structural features manifest in three-dimensional space, high-confidence models of the McACO proteins were generated (Figure S4). Compared to the extensive intrinsically disordered regions in the McACS family, McACO proteins displayed highly compact and rigid overall conformations. While minor, short terminal loops with lower confidence were observed in certain members (e.g., McACO1 and McACO3), the family largely lacked the substantial flexible extensions characteristic of ACS enzymes. Furthermore, the core regions of all five members exhibited exceptionally high pLDDT scores (>90). Structural superimposition revealed a strictly conserved jelly-roll β-barrel catalytic core. Magnified active site analysis of McACO2 confirmed the precise spatial arrangement of the canonical His-Asp-His catalytic triad (His178, Asp180, and His235) required for Fe(II) coordination (Figure 3d).

Taken together, these multi-dimensional analyses reveal a profound evolutionary dichotomy within the bitter gourd ethylene biosynthesis pathway. The upstream McACS enzymes exhibit substantial structural flexibility at their C-termini, associated with variable protein stability. Conversely, the downstream McACO enzymes rely on a rigid, highly conserved jelly-roll β-barrel scaffold, reflecting strict evolutionary constraints to ensure stable catalytic efficiency.

### 2.4. Chromosomal Distribution and Synteny Analysis

**Figure 4:**
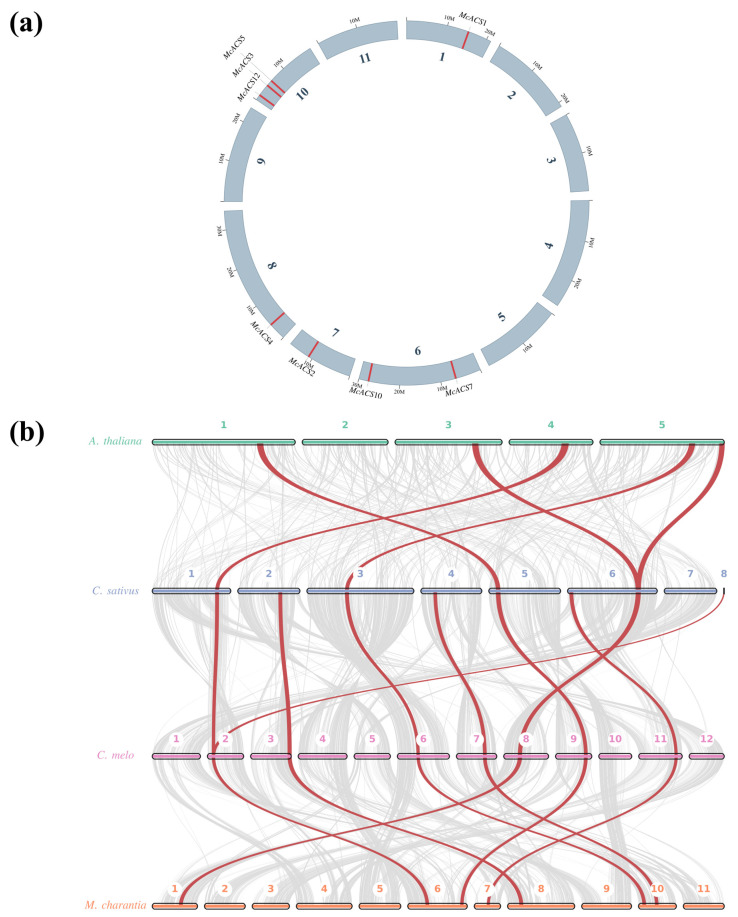
Chromosomal distribution and inter-species synteny of the *McACS* family. (**a**) Physical locations of the eight *McACS* genes on the 11 *M. charantia* chromosomes. (**b**) Syntenic relationships between *M. charantia* and reference species. Gray lines represent background collinear blocks, and colored lines indicate orthologous *McACS* gene pairs.

The chromosomal locations of the *McACS* and *McACO* genes were determined based on the Dali-11 chromosome-level reference genome. The eight *McACS* genes were distributed across five of the 11 chromosomes (Figure 4a): chromosome MC10 harbored three members (*McACS3*, *McACS5*, and *McACS12*), MC06 contained two (*McACS7* and *McACS10*), while MC01, MC07, and MC08 each contained a single member. Intra-species synteny analysis revealed a complete absence of duplication events within the *McACS* family, indicating high structural stability. Inter-species synteny analysis identified seven orthologous *McACS* pairs between *M. charantia* and reference cucurbit species (Figure 4b).

**Figure 5:**
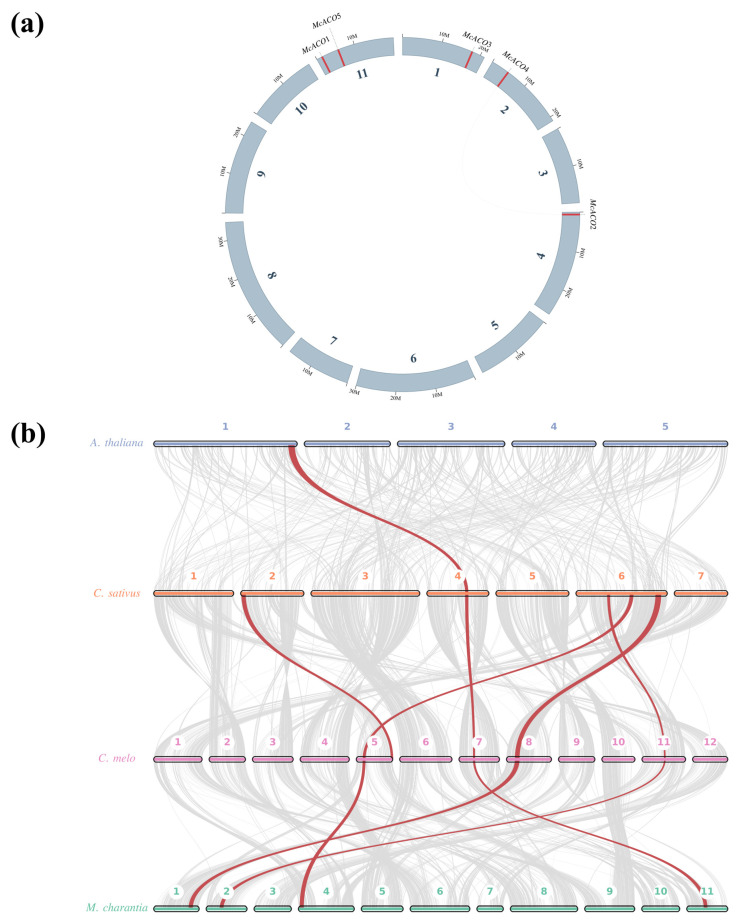
Chromosomal distribution and synteny analysis of the *McACO* family. (**a**) Chromosomal mapping of the five *McACO* genes. (**b**) Intra- and inter-species synteny analysis. The red arc indicates the segmental duplication event between *McACO2* and *McACO4*.

Similarly, the five *McACO* genes were mapped onto four chromosomes (Figure 5a). MC11 contained two genes (*McACO1* and *McACO5*), while MC01, MC02, and MC04 each harbored a single member (*McACO3*, *McACO4*, and *McACO2*, respectively). Intra-species synteny analysis identified one segmentally duplicated gene pair (*McACO2* on MC04 and *McACO4* on MC02), indicating that segmental duplication contributed to the limited expansion of the *McACO* family in bitter gourd (Figure 5b). No tandem duplication events were observed. Inter-species synteny analysis revealed five orthologous pairs for the *McACO* family within the Cucurbitaceae.

To further evaluate the evolutionary constraints acting on these two families, substitution rates were calculated for all identified orthologous and paralogous pairs (Table S3). The *Ka*/*Ks* ratios for the *McACS* and *McACO* families ranged from 0.038 to 0.084 and 0.040 to 0.105, respectively. These values are all strictly <1.0, demonstrating that both families have evolved under strong purifying selection. The estimated divergence times for the orthologous pairs within the Cucurbitaceae ranged from 10.86 to 66.26 million years ago (Mya), whereas the ancient segmental duplication event (*McACO2*/*McACO4*) was estimated to have occurred approximately 80.00 Mya.

### 2.5. Promoter Analysis and Transcriptomic Profiling

**Figure 6:**
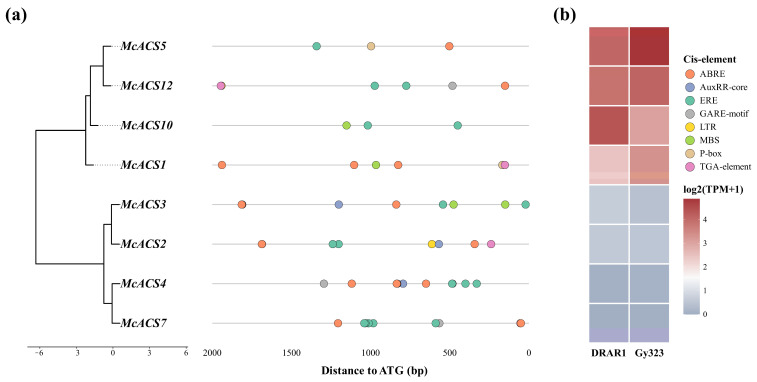
Integrated analysis of *McACS* promoter elements and expression patterns. (**a**) Distribution of cis-regulatory elements in the 2000 bp upstream regions of the eight *McACS* genes. Symbols indicate distinct hormone-, stress-, and light-responsive motifs. (**b**) Heatmap of absolute expression levels (TPM) for *McACS* genes in the strongly gynoecious line Gy323 and the monoecious accession DRAR1.

**Figure 7:**
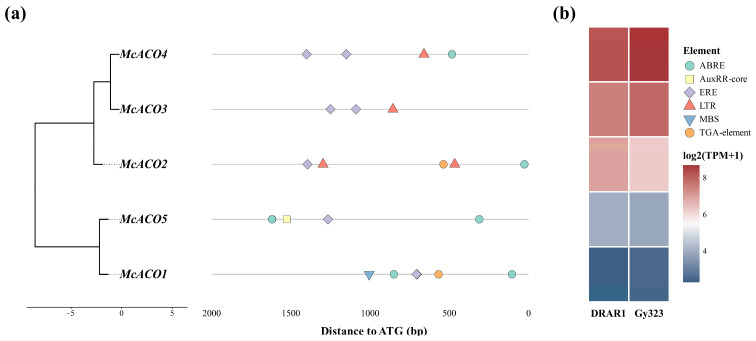
Integrated analysis of *McACO* promoter elements and expression patterns. (**a**) Distribution of cis-regulatory elements in the 2000 bp upstream regions of the five *McACO* genes. (**b**) Heatmap of absolute expression levels (TPM) for *McACO* genes in Gy323 and DRAR1.

To explore the regulatory mechanisms governing the ethylene biosynthesis genes, the 2000 bp upstream promoter regions of the *McACS* and *McACO* families were analyzed for cis-regulatory elements (Figure 6a and Figure 7a). Various hormone- and stress-responsive elements were identified, prominently including ethylene-responsive elements (ERE), abscisic acid-responsive elements (ABRE), methyl jasmonate-responsive motifs (CGTCA/TGACG), and auxin-responsive elements (TGA). Notably, the promoter of *McACS12* contained a high density of regulatory sites, including ABRE, ERE, TGA, and drought-inducible MBS motifs, while *McACS5* specifically featured MeJA- and anaerobic-responsive (ARE) elements. Similarly, *McACO3* and *McACO4* possessed multiple ERE and MeJA-responsive motifs, indicating their integration into complex hormonal and environmental signaling networks.

Transcriptional profiles were subsequently evaluated using public RNA-seq data to compare absolute expression levels (transcripts per million, TPM) between the strongly gynoecious line Gy323 and the monoecious accession DRAR1 (Figure 6b and Figure 7b). Within the *McACS* family, *McACS5* and *McACS12* emerged as the predominant members expressed in flower buds; specifically, *McACS5* exhibited substantially higher transcription in Gy323 (TPM = 28.29) than in DRAR1 (TPM = 15.66). Conversely, *McACS10* showed an accession-specific accumulation exclusively in DRAR1 (TPM = 19.80), while other typical members (*McACS2*, *McACS3*, *McACS4*, and *McACS7*) remained nearly silent (TPM < 1). In the *McACO* family, *McACO3* and *McACO4* displayed abundant transcript levels, with *McACO4* peaking dramatically in Gy323 (TPM = 410.51). The convergence of high transcript abundance and the presence of critical hormone-responsive elements highlights *McACS5*, *McACS12*, *McACO3*, and *McACO4* as potential functional candidates for mediating ethylene-dependent sex differentiation in *M. charantia*.

### 2.6. qRT-PCR Validation Under AgNO_3_ Treatment

It is noteworthy that the primary candidate genes identified from the public Gy323/DRAR1 RNA-seq dataset partially differ from those emphasized in our subsequent targeted qRT-PCR profiling. This discrepancy is likely attributed to the strong spatiotemporal specificity of ethylene biosynthetic genes. The public RNA-seq data capture a broader developmental snapshot, whereas our qRT-PCR analysis was strictly restricted to the critical 1.0–3.0 mm early floral bud stage under precise chemical induction. Therefore, the subsequent qRT-PCR results more accurately reflect the immediate transcriptional dynamics specific to this early developmental window.

**Figure 8:**
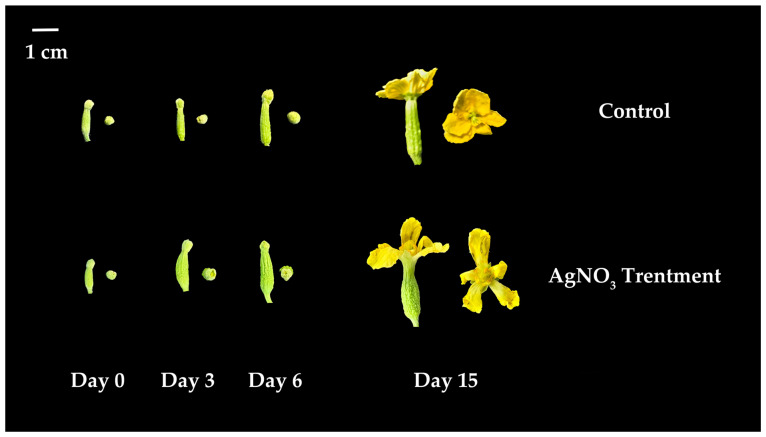
Morphological dynamics of AgNO_3_-induced sex reversal in the gynoecious bitter gourd line ‘2Y161’. Representative tracking photographs illustrate the continuous developmental progression of floral buds into mature open flowers at 0, 3, 6, and 15 days post-treatment under Control (distilled water) and AgNO_3_ (1 g L^−1^) treatment conditions. Macroscopic morphological divergence is clearly visible starting from Day 3 and Day 6 in the treatment group. By Day 15 (full-blooming stage), Control plants exhibit typical female flowers characterized by a normal green stigma and a distinct inferior ovary without stamens. Conversely, AgNO_3_-treated branches predominantly produce perfect hermaphroditic flowers, featuring fully developed, plicate yellow stamens surrounding the central stigma. (Scale bars are indicated directly on the panels; *n* = 30 individual plants evaluated per treatment group).

To evaluate the phenotypic changes induced by the chemical treatment, the morphological dynamics of floral buds in the gynoecious line ‘2Y161’ were documented from Day 0 to Day 15 (Figure 8). Under control conditions (distilled water), the evaluated plants (*n* = 30) developed standard female flowers, characterized by an inferior ovary and a central stigma without stamens at Day 15.

In contrast, plants treated with 1 g L^−1^ AgNO_3_ exhibited morphological divergence. Stamen-like structures were visually distinguishable in the developing buds at Day 3 and Day 6 post-treatment. At Day 15, the AgNO_3_-treated branches produced hermaphroditic flowers, which retained the basal ovary but developed yellow stamens around the central stigma. The morphological observations at Days 0, 3, and 6 correspond to the sampling stages used for the subsequent qRT-PCR analyses.

To validate the functional roles of the *McACS* and *McACO* families in sex determination, quantitative real-time PCR (qRT-PCR) was conducted using *HMG1/2* as the internal reference. Spatiotemporal expression dynamics were tracked during natural female flower differentiation (Day 0, Control-Day 3, and Control-Day 6) and under AgNO_3_ treatment (AgNO_3_-Day 3 and AgNO_3_-Day 6), which biochemically suppresses ethylene perception and induces male flower formation.

**Figure 9:**
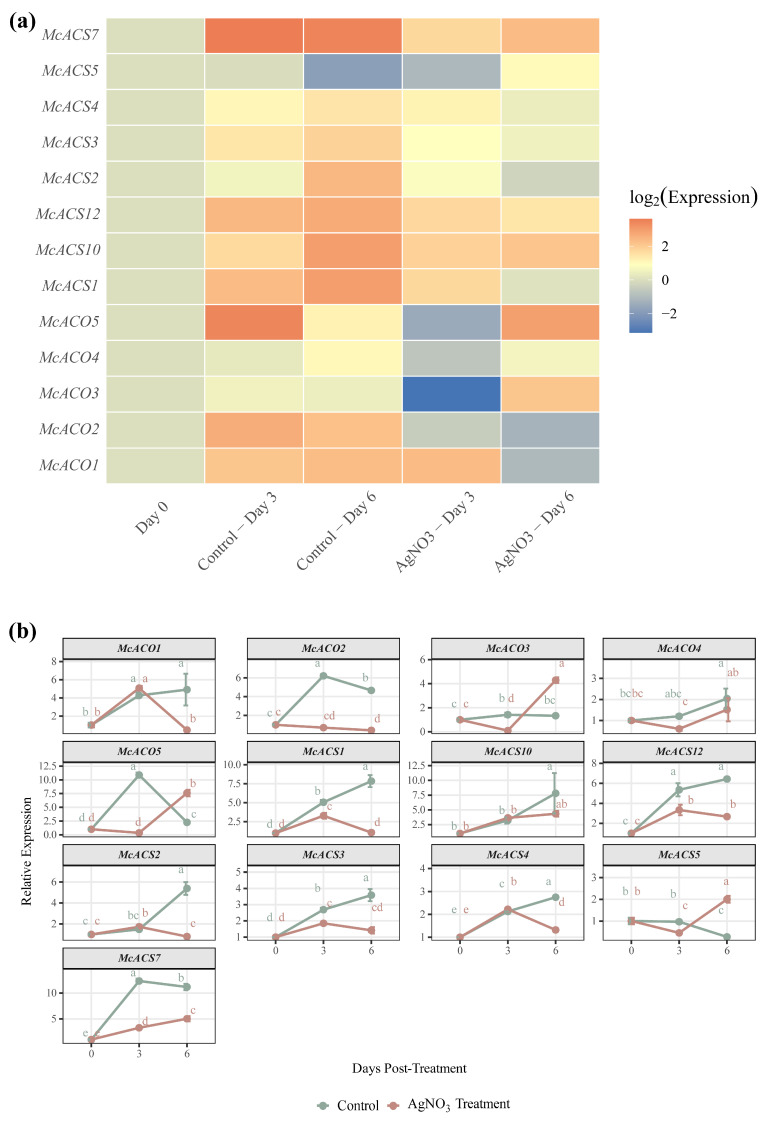
Transcriptional expression profiling of ethylene biosynthesis genes during natural flower development and AgNO_3_-induced sex reversal. (**a**) Heatmap of relative expression levels for the *McACS* and *McACO* genes across five developmental stages and treatments (Day 0, Control-Day 3, Control-Day 6, AgNO_3_-Day 3, and AgNO_3_-Day 6). The color scale indicates log_2_-transformed relative fold changes. (**b**) Expression trajectory plots of core candidate genes (*McACS1*, *McACS7*, *McACS12*, and *McACO2*) validated by qRT-PCR. Data are presented as the mean ± standard deviation (SD) of three independent biological replicates (*n* = 3), with each replicate dynamically pooled from 10 individual plants. Different lowercase letters (a, b, c, d, e) denote statistically significant differences according to Tukey’s HSD test (*p* < 0.05). Note: Independent *y*-axis scales were deliberately applied to individual trajectory panels in (**b**) because the absolute basal transcript abundance and induction magnitude vary massively across different gene family members; a unified scale would completely obscure the critical expression dynamics of genes with relatively lower baseline abundance.

Global expression patterns, visualized via a hierarchical clustering heatmap (Figure 9a), revealed distinct transcriptional profiles. During the natural developmental progression from Day 0 to Control-Day 6, a coordinated upregulation of multiple ethylene biosynthesis genes was observed, corresponding to a transcriptional induction essential for female flower organogenesis. Conversely, this comprehensive transcriptomic program was profoundly repressed in the AgNO_3_-treated groups.

To further resolve these expression dynamics, trajectory plots were generated for the core functional candidates (Figure 9b). Under natural control conditions, genes including *McACS1*, *McACS7*, *McACS12*, and *McACO2* exhibited a progressive upregulation; for instance, the transcript levels of *McACS7* and *McACS1* increased by approximately 15.4-fold and 10.8-fold at the Control-Day 6 stage, respectively. In contrast, the application of AgNO_3_ resulted in the profound suppression of these developmental expression peaks. The transcript levels of these candidate genes in the AgNO_3_-Day 3 and AgNO_3_-Day 6 groups remained near or below initial baseline levels (e.g., *McACO2* decreased to 0.42-fold relative to Day 0). This divergent expression trend indicates that chemically induced masculine organogenesis strictly coincides with the robust transcriptional repression of core ethylene biosynthetic genes.

## 3. Discussion

### 3.1. Genomic Identification and Evolutionary Dynamics

The accurate identification of gene families is fundamentally reliant on the completeness of the reference genome [17]. Early investigations into ethylene biosynthesis in *M. charantia* predominantly utilized cDNA cloning strategies to isolate fragmented gene sequences [18,19]. Constrained by the absence of whole-genome data, these initial reports yielded incomplete family profiles and nomenclature redundancies. In this study, eight *McACS* and five *McACO* genes were systematically identified utilizing the chromosome-level Dali-11 genome [20], thereby standardizing their nomenclature and updating previous annotations.

Evolutionary analyses indicated that while both families underwent strong purifying selection [21], they exhibited distinct evolutionary trajectories. The *McACO* family demonstrated high sequence conservation, functioning as a rigid catalytic core [22], with a specific segmental duplication event (*McACO2*/*McACO4*) likely serving to expand its functional dosage [23]. In contrast, the *McACS* family displayed extensive structural diversity, reflecting varied potential regulatory capacities [6,24]. This divergence suggests a sophisticated evolutionary adaptation: conserving the downstream enzymatic step while diversifying the upstream regulatory components [6]. This bipartite mechanism presumably enables *M. charantia* to fine-tune ethylene biosynthesis precisely during critical developmental processes, particularly organogenesis and floral development.

### 3.2. Structural Differences and Potential Regulatory Roles of McACS Proteins

While the catalytic cores of ACS enzymes are highly conserved, their post-translational regulation is primarily governed by their C-terminal domains [24]. AlphaFold 3-based structural modeling revealed substantial conformational heterogeneity within the McACS family, consistent with their phylogenetic divergence. Proteins, including the basal (McACS1), Type I (McACS2/5), Type II (McACS3/4), and non-canonical (McACS10/12) members, possess extended, intrinsically disordered C-terminal regions. In model plant species, these unstructured extensions serve as target degrons for ubiquitin-mediated proteasomal degradation, facilitating rapid protein turnover [9,10]. In stark contrast, the sole Type III member, McACS7, adopts a highly compact conformation completely lacking this flexible extension.

Expression profiling demonstrated that *McACS7*—alongside the non-canonical *McACS12*—was substantially upregulated during natural female flower differentiation. The preferential recruitment of the structurally rigid McACS7 during this process suggests a distinct functional adaptation. Devoid of typical C-terminal degradation targets, McACS7 likely evades rapid proteasomal clearance, thereby potentially offering greater enzymatic stability to support the sustained ethylene production associated with female floral development. This structural dichotomy provides a plausible physical basis for why specific *McACS* paralogs, particularly the Type III isoform, are selectively co-opted to orchestrate prolonged developmental processes.

### 3.3. Transcriptional Dynamics of Ethylene Biosynthesis Genes and Response to AgNO_3_

In Cucurbitaceae, local ethylene accumulation serves as a primary factor associated with female flower development [25]. As demonstrated by qRT-PCR, the transcript levels of specific candidates—namely *McACS1*, *McACS7*, *McACS12*, and *McACO2*—were progressively upregulated during natural floral bud development (from Day 0 to Control-Day 6). This developmental induction aligns with the critical window for establishing gynoecy. Conversely, AgNO_3_ functions as a potent ethylene perception inhibitor routinely utilized to induce male or hermaphroditic flowers [11]. Following AgNO_3_ application (AgNO_3_-Day 3 and AgNO_3_-Day 6), the expression of these biosynthetic genes was strictly restricted to basal levels.

The transcriptional suppression of ethylene biosynthesis genes by a perception inhibitor aligns with previous hypotheses suggesting an autocatalytic positive feedback loop in cucurbit sex determination [13]. By physically blocking ethylene receptors, AgNO_3_ disrupts downstream signaling cascades, which in our study coincided with the prevention of the transcriptional activation of the biosynthetic machinery. This uncoupling likely restricts autogenous ethylene production, ultimately diverting organogenesis toward male development. However, we acknowledge that the present evidence relies strictly on transcript-level responses. Due to the extremely low biomass of the 1.0–3.0 mm early floral buds, precise quantification of endogenous ACC content, ethylene gas emission, and ACS/ACO enzymatic activities was not feasible in the current experimental setup. Further biochemical validations are required to comprehensively elucidate the precise execution of this regulatory loop in *M. charantia*.

### 3.4. Comparative Sex Determination and Future Perspectives

In the model plant *C. sativus*, specific *ACS* genes corresponding to the *F* (*CsACS1G*), *M* (*CsACS2*), and *A* (*CsACS11*) loci rigorously control sex differentiation [3,4]. Recent genetic mappings in *M. charantia* have localized gynoecious loci (e.g., *Mcgy1*) to Chromosome 1, proposing atypical upstream regulators, such as *CTPS*, rather than classical ethylene biosynthesis genes [15,16]. This genomic divergence suggests that *M. charantia* utilizes a distinct initial genetic trigger. Nevertheless, because the physiological basis of feminization across Cucurbitaceae fundamentally relies on ethylene, downstream biosynthetic effectors remain biologically indispensable.

The current findings offer potential downstream candidates to complement this regulatory model. While upstream loci like *CTPS* may initiate the developmental signal, structural predictions and expression dynamics indicate that the actualization of the female phenotype correlates with the recruitment of structurally rigid paralogs, notably *McACS7* and *McACS12*, to execute the localized ethylene surge. Because the present study primarily relies on in silico identification and transcriptional profiles without direct in vivo functional validation, the precise biological roles of these candidate genes require further elucidation. Nonetheless, these genome-wide findings establish a comprehensive theoretical framework for understanding ethylene-associated floral development in *M. charantia*. Future experimental investigations employing targeted genetic manipulation, such as CRISPR/Cas9-mediated knockout or overexpression, are necessary to ascertain their specific physiological phenotypes. Ultimately, such functional validations will yield reliable reference targets for the molecular breeding of stable gynoecious lines.

Previous studies on sex determination in Cucurbitaceae primarily focused on traditional genetic mapping [15,16] or fragment cloning [18,19]. In this study, we integrated the chromosome-level reference genome (Dali-11) with AlphaFold 3-based structural predictions to provide a more comprehensive perspective. Beyond evolutionary comparative genomics, our work explores the potential biological functions of these genes. Specifically, the variation in intrinsically disordered C-terminal extensions—such as their absence in McACS7—may affect enzyme stability and protein degradation during floral transitions. Furthermore, this study connects structural divergence with transcriptional regulation under chemical induction (AgNO_3_). Overall, these findings improve our understanding of the molecular mechanisms underlying sex determination in *M. charantia* and offer candidate functional targets for the marker-assisted breeding of gynoecious lines, which is relevant for agricultural yield.

## 4. Materials and Methods

### 4.1. Plant Materials and AgNO_3_ Treatments

The gynoecious *M. charantia* line ‘2Y161’ was cultivated under standard field conditions at the experimental station of the Tropical Eco-agriculture Research Institute, Yunnan Academy of Agricultural Sciences (Yuanmou, Yunnan, China). The experimental plot followed a completely randomized design with 30 individual plants per treatment group. For chemical treatments, an aqueous solution of 1 g L^−1^ AgNO_3_ was thoroughly sprayed onto seedlings at the three-leaf stage until runoff (approximately 15 mL per plant), followed by a secondary targeted application to emerging lateral branches prior to flowering. The concentration of 1 g L^−1^ was selected based on our long-term empirical agricultural practices and field breeding experience, which consistently proved optimal for inducing sex reversal in this gynoecious line without introducing severe phytotoxicity. The solution was applied directly without the addition of any surfactants. Control plants were mock-treated with an equivalent volume of double-distilled water.

To systematically trace the progression of sex reversal, close-up digital photography was performed to document the macroscopic morphological dynamics of developing floral buds from both groups at 0, 3, and 6 days post-treatment. Concurrently, equivalent developing floral buds (strictly 1.0–3.0 mm in size, representing the critical early window of sex determination) were harvested from the identical time points. All molecular samples were pooled from three biological replicates, immediately frozen in liquid nitrogen, and stored at −80 °C until RNA extraction and subsequent qRT-PCR analysis.

### 4.2. Genome-Wide Identification of ACS and ACO Genes

The chromosome-level genome assembly of *M. charantia* ‘Dali-11’ [20] was used to identify gene family members. Homologous sequences in *M. charantia*, *A. thaliana*, *C. sativus*, and *C. melo* were identified using Hidden Markov Model (HMM) profiles (PF00155 for ACS; PF03171 for ACO) obtained from the Pfam database [26] via hmmsearch [27]. Local BLASTP alignments (E-value < 1 × 10^−5^) were simultaneously performed [28]. Candidates were filtered by sequence length (400–600 aa for ACS; 250–450 aa for ACO), verified via the NCBI Conserved Domain Database (CDD) [29], and required to share >40% amino acid sequence identity with *A. thaliana* reference sequences. Physicochemical properties, including molecular weight and isoelectric point, were calculated using the Biopython package [30]. The subcellular localization was predicted using WoLF PSORT (https://wolfpsort.hgc.jp/, accessed on 15 May 2026), with the organism set to “Plant”.

### 4.3. Sequence Alignment, Phylogeny, and Motif Analysis

Full-length protein sequences were aligned using MAFFT [31]. Maximum likelihood (ML) phylogenetic trees were constructed using IQ-TREE [32], with optimal substitution models determined by ModelFinder [33] and branch support assessed via 1000 ultrafast bootstraps [34]. Conserved motifs were predicted using the MEME suite [35], with the maximum number of motifs set to 10. Gene structures (CDS and UTRs) were mapped from the corresponding GFF3 annotation files. The integration of phylogenetic relationships, conserved motifs, and gene structures was visualized using the R packages ggtree [36] and ggplot2 [37].

### 4.4. Chromosomal Synteny and Evolutionary Dynamics

Genomic synteny analysis was performed using MCScanX [38] to detect collinear blocks within and between species. Prior to analysis, all-vs-all BLASTp searches (https://blast.ncbi.nlm.nih.gov/, accessed on 15 May 2026) were executed with an E-value threshold of <1 × 10^−5^ to identify homologous protein pairs. The resulting collinearity data were visualized using the Python package pycirclize (version 1.10.1, https://github.com/moshi4/pycirclize, accessed on 15 May 2026). To evaluate the evolutionary selection pressure acting on the McACS and McACO gene families, the coding sequences (CDS) of identified orthologous and paralogous gene pairs were aligned using the MUSCLE algorithm. The non-synonymous (Ka) and synonymous (Ks) substitution rates were calculated using KaKs_Calculator 2.0 with the YN (Yang-Nielsen) model [39]. Divergence times (T) were estimated using the formula T = Ks/(2λ), adopting a standard dicotyledonous mutation rate (λ) of 1.5 × 10^−8^ substitutions site^−1^ year^−1^ [40]. Tandem duplication events were defined as gene pairs located within a 100 kb genomic window showing physical linkage on the same chromosome, while all other collinear pairs were classified as segmental duplications.

### 4.5. Promoter Analysis and RNA-Seq Expression Profiling

To identify *cis*-acting regulatory elements, 2000 bp sequences upstream of the start codons were extracted and analyzed using the PlantCARE database [41]. For expression profiling, public *M. charantia* RNA-seq datasets (accession numbers: SRR947759, SRR953077, SRR950973, and SRR953078), which were originally reported by Shukla et al. [42], were utilized. Raw reads were processed using fastp [43] and aligned to the reference genome via HISAT2 [44]. Resulting alignments were sorted and converted to BAM format using SAMtools [45], and gene-level abundances were quantified with featureCounts [46]. Expression levels were log_2_-transformed to generate clustered heatmaps for visualization.

### 4.6. RNA Extraction and qRT-PCR Validation

Total RNA was extracted using the Plant Total RNA Kit (Magen, Guangzhou, China) and reverse-transcribed using the FastKing RT Kit (Biosharp, Beijing, China). To ensure data reliability, the expression stability of five candidate reference genes was evaluated via RefFinder [47], and *HMG1/2* was selected as the internal reference due to its highest stability ranking across all algorithms (Table S4). The qRT-PCR assays were performed on a LightCycler 96 System (Roche, Basel, Switzerland) using the SYBR Green qRT-PCR Mix (Biosharp, Beijing, China). Relative expression levels were calculated utilizing the 2^−ΔΔCt^ method [48]. Specific primer sequences are detailed in Table S1.

### 4.7. Protein 3D Structure Modeling

The 3D structures of *M. charantia* ACS and ACO proteins were predicted using AlphaFold 3 [49]. The predicted local distance difference test (pLDDT) scores were used to evaluate model confidence. Structural alignments, surface mapping, and the visualization of active site pockets (catalytic triads) were performed using PyMOL v3.1.0 [50].

## 5. Conclusions

In conclusion, this study identified eight *McACS* and five *McACO* genes in *M. charantia* using the chromosome-level Dali-11 genome. Evolutionary and 3D structural analyses revealed distinct structural features between the two families: McACO proteins maintain a rigid and highly conserved catalytic core, whereas McACS members display diverse C-terminal flexibilities associated with their predicted protein stabilities. Targeted expression profiling during the critical 1.0–3.0 mm early floral bud stage demonstrated that specific structurally stable Type III McACS members, notably *McACS7*, are significantly upregulated during natural female flower development. Furthermore, the robust transcriptional suppression of these core biosynthetic genes by the perception inhibitor AgNO_3_ provides compelling candidate-gene evidence linking the local ethylene biosynthetic machinery to chemically induced sex reversal. Overall, rather than fully elucidating the downstream molecular mechanisms, these findings characterize the structural determinants and expression dynamics of ethylene biosynthesis genes in bitter gourd, offering valuable reference targets for future functional validations and the molecular breeding of stable gynoecious lines.

## Figures and Tables

**Table 1:** Characteristics of the ACS and ACO gene family members identified in *M. charantia*.

Symbol	Original ID	Length (aa)	MW (kDa)	pI	Subcellular
*McACS1*	MC01g0962	480	54.21	8.95	Nucleus
*McACS2*	MC07g0325	495	55.49	6.14	Nucleus
*McACS3*	MC10g0527	460	52.01	8.6	Chloroplast
*McACS4*	MC08g0531	433	48.73	6.39	Nucleus
*McACS5*	MC10g0702	484	54.13	5.99	Cytoplasm
*McACS7*	MC06g0753	447	50.05	5.57	Cytoplasm
*McACS10*	MC06g2011	548	60.17	6.98	Chloroplast
*McACS12*	MC10g0187	510	56.61	8.34	Plasma membrane
*McACO1*	MC11g0190	298	33.92	5.49	Cytoplasm
*McACO2*	MC04g0085	317	35.83	5.43	Cytoskeleton
*McACO3*	MC01g1295	322	36.58	5.23	Cytoplasm
*McACO4*	MC02g0580	319	36.14	5.41	Cytoplasm
*McACO5*	MC11g0717	309	34.92	5.17	Cytoplasm

## Data Availability

The original contributions presented in the study are included in the article and Supplementary Materials. The publicly available chromosome-level Dali-11 reference genome of bitter melon analyzed in this study can be accessed via the China National GeneBank Database (CNGBdb) under project accession number CNP0000016 (BioProject: PRJEB24032). Further inquiries can be directed to the corresponding author.

## Supplementary Materials

The following supporting information can be downloaded at: https://www.mdpi.com/article/10.3390/ijms27135980/s1.
