# Supplementary material for: Structural Characterization and Expression Profiling of Ethylene Biosynthetic Genes During AgNO3-Induced Sex Reversal in Bitter Gourd
Source: Int J Mol Sci. 2026 Jul 3;27(13):5980. doi: 10.3390/ijms27135980 (PMC13362086; doi:10.3390/ijms27135980)
Supplement: Supplementary file 1 [file ijms-27-05980-s001.zip › ijms-4341219-supplementary.pdf]

Table S1. The primer sequences used in the experiment

Genes	Purpose	Primer sequence (5′-3′)	Length（bp）	Gene ID / Accession No.
**Target Genes**				
McACS1	Expression analysis	F: CCAAAAACCCAGATGCAGCC<br>R: AGCACGATGTTGTCAGGGTT	169	MC01g0962
McACS2	Expression analysis	F: GAGGGGTGGGAGAGTGAGAT<br>R: GGGATCGGCCAAACAGAAGA	97	MC07g0325
McACS3	Expression analysis	F: CTCCCCGCATTCAAAAAGGC<br>R: ATGGGGTAGGCAGGAGGAAT	169	MC10g0527
McACS4	Expression analysis	F: CGGATCCGGAAGAGAAAGGG<br>R: CCGAACCGGGGGAGATATTG	193	MC08g0531
McACS5	Expression analysis	F: GGCACAGTTTACGACAGGGA<br>R: GCGTAGATTTCGTCGCACAC	89	MC10g0702
McACS7	Expression analysis	F: ATTGAAGGATTGCGAACCGC<br>R: GGTTCAGAGCAGTGACACGA	188	MC06g0753
McACS10	Expression analysis	F: CTCCGGACCCGAAATTCCAA<br>R: CCCTGCAGAGGCACTATGAC	95	MC06g2011
McACS12	Expression analysis	F: GTGGATCCATTGGACGGGTT<br>R: CCTGTTCTCGGACAACCCAA	161	MC10g0187
McACO1	Expression analysis	F: AAGTCGCGAAATACCCGGAA<br>R: GAATCTTCAACCACACGCCG	147	MC11g0190
McACO2	Expression analysis	F: CAATTACCCACCGTGTCCGA<br>R: CCTCAAGCTGATCCCCAAGG	188	MC04g0085
McACO3	Expression analysis	F: TATCGCACGAGCTGATGGAC<br>R: GGAAGAACGTGCTCTCCCAA	153	MC01g1295
McACO4	Expression analysis	F: GTTCCAAGAGCTCGTGTCCA<br>R: GTTGGAGACTGGGAGATGGC	106	MC02g0580
McACO5	Expression analysis	F: GCGGTGAGAAGCTGGAGAAT<br>R: GCTCCGATCTGTACTCTGCC	117	MC11g0717
**Reference Genes**				
GAPDH	Ref. screening	F: CCACCAACTGTCTTGCTCCA<br>R: ACAGTCTTCTGGGTTGCAGTA	106	XM_022278270.1
PHOS32	Ref. screening	F: AACCACCGGATCTCCGCTTA<br>R: TGCAGTCGTCACAATGTCCA	109	XM_022282583.1
TIP41	Ref. screening	F: AGCAAACCTTCCCAACAGGT<br>R: TGCTCTCCTTCATGCTTCTCTG	107	XM_022293162.1
HMG1/2	Ref. screening	F: TTCGGTTCCCCTTTCCTCAC<br>R: TCATGTCGAATCCTCGGTGG	105	XM_022286896.1
ACT7	Ref. screening	F: CACAGCCACACCTTCGTCTT<br>R: CAGCAAAGCCAGCCTTCAC	137	XM_022277165.1

Table S2. Physicochemical properties of *ACS* and *ACO* gene families in four plant species

Symbol	Original_ID	Length_aa	MW_kDa	pI	Subcellular
McACS1	MC01g0962	480	54.21	8.95	Cytoplasm
McACS7	MC06g0753	447	50.05	5.57	Cytoplasm
McACS10	MC06g2011	548	60.17	6.98	Cytoplasm
McACS2	MC07g0325	495	55.49	6.14	Cytoplasm
McACS4	MC08g0531	433	48.73	6.39	Cytoplasm
McACS12	MC10g0187	510	56.61	8.34	Cytoplasm
McACS3	MC10g0527	460	52.01	8.60	Cytoplasm
McACS5	MC10g0702	484	54.13	5.99	Cytoplasm
CsACS7-like	XP_031738416.1	444	50.33	5.62	Nucleus
CsACS3-like	XP_004142909.2	441	49.92	5.99	Nucleus
CsACS12	XP_004148155.1	510	56.56	8.50	Plasma membrane
CsACS-like	NP_001267589.1	489	54.78	6.29	Cytoplasm
CsACS3	XP_004137286.1	442	49.72	8.55	Cytoplasm
CsACS10	XP_004147561.1	548	60.37	6.00	Chloroplast
CsACS2	NP_001292642.1	493	55.51	6.55	Nucleus
CsACS1G	NP_001295859.1	481	54.22	8.42	Nucleus
CsACS7	NP_001292629.1	445	50.42	5.62	Nucleus
CmACS7	NP_001315384.1	445	50.47	5.83	Nucleus
CmACS9	XP_008445556.3	452	51.81	8.00	Nucleus
CmACS12	XP_008439119.1	510	56.56	8.50	Plasma membrane
CmACS-like	XP_008452008.2	490	54.90	6.11	Cytoplasm
CmACS1G	NP_001284463.2	481	54.25	8.45	Nucleus
CmACS10	XP_008437168.1	548	60.39	5.68	Chloroplast
CmACS1	NP_001284464.1	493	55.61	6.39	Nucleus
AtACS2	NP_171655.1	496	55.53	7.20	Cytoplasm
AtACS10	NP_564804.1	557	61.02	6.69	Chloroplast
AtACS4	NP_179866.1	474	53.79	8.50	Nucleus
AtACS9	NP_190539.1	470	53.17	6.73	Nucleus
AtACS1	NP_191710.1	488	55.00	7.18	Nucleus
AtACS11	NP_567330.1	460	51.80	6.34	Nucleus
AtACS6	NP_192867.1	495	55.52	6.23	Cytoplasm
AtACS7	NP_194350.1	447	50.66	5.94	Cytoplasm
AtACS8	NP_195491.1	469	53.37	8.00	Nucleus
AtACS12	NP_199982.2	495	55.21	7.03	Chloroplast
AtACS5	NP_201381.1	470	53.31	7.55	Nucleus
AtACO1	NP_179549.1	310	35.20	6.17	Cytoplasm
AtACO2	NP_176428.1	320	36.18	4.98	Cytoplasm
AtACO3	NP_171994.1	323	36.68	5.24	Cytoplasm
AtACO4	NP_172665.1	320	36.53	5.09	Cytoplasm
AtACO5	NP_565154.1	307	34.95	5.05	Cytoskeleton
CmACO1	XP_008459274.1	300	34.22	5.95	Peroxisome
CmACO2	NP_001284392.1	318	36.13	5.26	Cytoplasm
CmACO3	XP_008440054.1	320	36.40	5.12	Cytoplasm
CmACO4	XP_008456405.1	314	35.46	5.25	Cytoplasm

Symbol	Original_ID	Length_aa	MW_kDa	pI	Subcellular
CmACO5	XP_008444197.2	309	34.91	5.07	Cytoplasm
CsACO1	XP_031736607.1	306	34.87	6.27	Peroxisome
CsACO2	NP_001267694.1	317	35.86	5.26	Cytoplasm
CsACO3	NP_001292672.1	317	36.12	5.40	Cytoplasm
CsACO4	NP_001295778.1	314	35.37	5.17	Cytoplasm
CsACO5	XP_004142637.1	309	34.90	5.19	Cytoplasm
McACO1	MC11g0190	298	33.92	5.49	Cytoplasm
McACO2	MC04g0085	317	35.83	5.43	Cytoskeleton
McACO3	MC01g1295	322	36.58	5.23	Cytoplasm
McACO4	MC02g0580	319	36.14	5.41	Cytoplasm
McACO5	MC11g0717	309	34.92	5.17	Cytoplasm

Table S3. Ka/Ks analysis and estimated divergence times of sytenic *ACS* and *ACO* gene pairs.

Sequence_Pair	Gene1	Gene2	Homology_Type	Ka	Ks	Ka/Ks	Selection_Pressure	Divergence_Time_MYA
*McACS1*_vs_*AtACS8*	*McACS1*	*AtACS8*	Synteny (Interspecific)	0.173736	4.384530	0.039625	Purifying Selection	146.15
*McACS1*_vs_*AtACS5*	*McACS1*	*AtACS5*	Synteny (Interspecific)	0.174598	4.356530	0.040077	Purifying Selection	145.22
*McACS1*_vs_*CsACS1G*	*McACS1*	*CsACS1G*	Synteny (Interspecific)	0.063331	0.999941	0.063335	Purifying Selection	33.33
*McACS1*_vs_*CmACS1G*	*McACS1*	*CmACS1G*	Synteny (Interspecific)	0.058461	1.134950	0.051510	Purifying Selection	37.83
*McACS7*_vs_*CsACS7-like*	*McACS7*	*CsACS7-like*	Synteny (Interspecific)	0.058643	1.018200	0.057594	Purifying Selection	33.94
*McACS10*_vs_*CsACS10*	*McACS10*	*CsACS10*	Synteny (Interspecific)	0.082348	0.361308	0.227916	Purifying Selection	12.04
*McACS7*_vs_*CmACS7*	*McACS7*	*CmACS7*	Synteny (Interspecific)	0.057792	0.962056	0.060071	Purifying Selection	32.07
*McACS10*_vs_*CmACS10*	*McACS10*	*CmACS10*	Synteny (Interspecific)	0.084739	0.363725	0.232976	Purifying Selection	12.12
*McACS7*_vs_*CsACS7*	*McACS7*	*CsACS7*	Synteny (Interspecific)	0.059832	1.011740	0.059137	Purifying Selection	33.72
*McACS2*_vs_*AtACS1*	*McACS2*	*AtACS1*	Synteny (Interspecific)	0.261096	4.395020	0.059407	Purifying Selection	146.5
*McACS2*_vs_*CsACS2*	*McACS2*	*CsACS2*	Synteny (Interspecific)	0.094822	0.957536	0.099027	Purifying Selection	31.92
*McACS2*_vs_*CmACS1*	*McACS2*	*CmACS1*	Synteny (Interspecific)	0.089046	0.925671	0.096196	Purifying Selection	30.86

**Sequence_Pair**	**Gene1**	**Gene2**	**Homology_Type**	**Ka**	**Ks**	**Ka/Ks**	**Selection_Pressure**	**Divergence_Time_MYA**
*McACS4*_vs_*CsACS3-like*	*McACS4*	*CsACS3-like*	Synteny (Interspecific)	0.168387	3.240370	0.051965	Purifying Selection	108.01
*McACS4*_vs_*CmACS9*	*McACS4*	*CmACS9*	Synteny (Interspecific)	0.161079	1.733960	0.092897	Purifying Selection	57.8
*McACS5*_vs_*AtACS6*	*McACS5*	*AtACS6*	Synteny (Interspecific)	0.265858	4.452220	0.059714	Purifying Selection	148.41
*McACS12*_vs_*CsACS12*	*McACS12*	*CsACS12*	Synteny (Interspecific)	0.032451	0.354149	0.091631	Purifying Selection	11.8
*McACS5*_vs_*CsACS-like*	*McACS5*	*CsACS-like*	Synteny (Interspecific)	0.046055	1.431750	0.032167	Purifying Selection	47.73
*McACS3*_vs_*CsACS3*	*McACS3*	*CsACS3*	Synteny (Interspecific)	0.095806	3.723270	0.025732	Purifying Selection	124.11
*McACS12*_vs_*CmACS12*	*McACS12*	*CmACS12*	Synteny (Interspecific)	0.033362	0.360952	0.092428	Purifying Selection	12.03
*McACS5*_vs_*CmACS-like*	*McACS5*	*CmACS-like*	Synteny (Interspecific)	0.046796	1.544710	0.030295	Purifying Selection	51.49
*AtACS10*_vs_*CsACS10*	*AtACS10*	*CsACS10*	Synteny (Interspecific)	0.295071	1.979930	0.149030	Purifying Selection	66
*AtACS10*_vs_*CmACS10*	*AtACS10*	*CmACS10*	Synteny (Interspecific)	0.301479	1.874680	0.160817	Purifying Selection	62.49
*AtACS4*_vs_*AtACS8*	*AtACS4*	*AtACS8*	Segmental Duplication (Intraspecific)	0.114230	1.047520	0.109049	Purifying Selection	34.92
*AtACS4*_vs_*CmACS1G*	*AtACS4*	*CmACS1G*	Synteny (Interspecific)	0.213375	4.350590	0.049045	Purifying Selection	145.02
*AtACS9*_vs_*AtACS5*	*AtACS9*	*AtACS5*	Segmental Duplication (Intraspecific)	0.044658	0.845326	0.052829	Purifying Selection	28.18

**Sequence_Pair**	**Gene1**	**Gene2**	**Homology_Type**	**Ka**	**Ks**	**Ka/Ks**	**Selection_Pressure**	**Divergence_Time_MYA**
*AtACS9*_vs_*CsACS1G*	*AtACS9*	*CsACS1G*	Synteny (Interspecific)	0.159138	4.311140	0.036913	Purifying Selection	143.7
*AtACS9*_vs_*CmACS1G*	*AtACS9*	*CmACS1G*	Synteny (Interspecific)	0.160179	4.302220	0.037232	Purifying Selection	143.41
*AtACS1*_vs_*CmACS1*	*AtACS1*	*CmACS1*	Synteny (Interspecific)	0.219464	4.378630	0.050122	Purifying Selection	145.95
*AtACS7*_vs_*CsACS7-like*	*AtACS7*	*CsACS7-like*	Synteny (Interspecific)	0.173696	4.345990	0.039967	Purifying Selection	144.87
*AtACS7*_vs_*CmACS7*	*AtACS7*	*CmACS7*	Synteny (Interspecific)	0.175641	4.343840	0.040435	Purifying Selection	144.79
*AtACS5*_vs_*CsACS1G*	*AtACS5*	*CsACS1G*	Synteny (Interspecific)	0.168445	4.334370	0.038863	Purifying Selection	144.48
*AtACS5*_vs_*CmACS1G*	*AtACS5*	*CmACS1G*	Synteny (Interspecific)	0.168521	4.330780	0.038912	Purifying Selection	144.36
*CsACS7-like*_vs_*CmACS7*	*CsACS7-like*	*CmACS7*	Synteny (Interspecific)	0.008255	0.135624	0.060870	Purifying Selection	4.52
*CsACS7-like*_vs_*CsACS7*	*CsACS7-like*	*CsACS7*	Segmental Duplication (Intraspecific)	NA	0.009584	0.000000	Purifying Selection	0.32
*CsACS3-like*_vs_*CmACS9*	*CsACS3-like*	*CmACS9*	Synteny (Interspecific)	0.051715	0.227071	0.227749	Purifying Selection	7.57
*CsACS12*_vs_*CmACS12*	*CsACS12*	*CmACS12*	Synteny (Interspecific)	0.005126	0.071839	0.071357	Purifying Selection	2.39
*CsACS-like*_vs_*CmACS-like*	*CsACS-like*	*CmACS-like*	Synteny (Interspecific)	0.010497	0.127739	0.082175	Purifying Selection	4.26
*CsACS10*_vs_*CmACS10*	*CsACS10*	*CmACS10*	Synteny (Interspecific)	0.014033	0.050672	0.276935	Purifying Selection	1.69

**Sequence_Pair**	**Gene1**	**Gene2**	**Homology_Type**	**Ka**	**Ks**	**Ka/Ks**	**Selection_Pressure**	**Divergence_Time_MYA**
*CsACS1G*_vs_*CmACS1G*	*CsACS1G*	*CmACS1G*	Synteny (Interspecific)	0.008984	0.085685	0.104844	Purifying Selection	2.86
*CsACS2*_vs_*CmACS1*	*CsACS2*	*CmACS1*	Synteny (Interspecific)	0.017606	0.119106	0.147816	Purifying Selection	3.97
*CmACS7*_vs_*CsACS7*	*CmACS7*	*CsACS7*	Synteny (Interspecific)	0.008276	0.134300	0.061622	Purifying Selection	4.48
*McACO3*_vs_*AtACO2*	*McACO3*	*AtACO2*	Ortholog (Interspecific)	0.222259	4.037020	0.055055	Purifying Selection	134.57
*McACO3*_vs_*AtACO4*	*McACO3*	*AtACO4*	Ortholog (Interspecific)	0.208957	4.061090	0.051453	Purifying Selection	135.37
*McACO3*_vs_*CsACO3*	*McACO3*	*CsACO3*	Ortholog (Interspecific)	0.080556	1.987880	0.040523	Purifying Selection	66.26
*McACO3*_vs_*CmACO3*	*McACO3*	*CmACO3*	Ortholog (Interspecific)	0.074114	1.410710	0.052537	Purifying Selection	47.02
*McACO4*_vs_*McACO2*	*McACO4*	*McACO2*	Paralog (Intraspecific)	0.116425	2.399890	0.048513	Purifying Selection	80
*McACO4*_vs_*CsACO4*	*McACO4*	*CsACO4*	Ortholog (Interspecific)	0.112327	3.987020	0.028173	Purifying Selection	132.9
*McACO4*_vs_*CmACO4*	*McACO4*	*CmACO4*	Ortholog (Interspecific)	0.105507	4.027970	0.026194	Purifying Selection	134.27
*McACO2*_vs_*CsACO2*	*McACO2*	*CsACO2*	Ortholog (Interspecific)	0.052145	0.541279	0.096336	Purifying Selection	18.04
*McACO2*_vs_*CmACO2*	*McACO2*	*CmACO2*	Ortholog (Interspecific)	0.049760	0.475341	0.104683	Purifying Selection	15.84
*McACO5*_vs_*AtACO5*	*McACO5*	*AtACO5*	Ortholog (Interspecific)	0.159735	1.803570	0.088566	Purifying Selection	60.12

**Sequence_Pair**	**Gene1**	**Gene2**	**Homology_Type**	**Ka**	**Ks**	**Ka/Ks**	**Selection_Pressure**	**Divergence_Time_MYA**
*McACO5*_vs_*CsACO5*	*McACO5*	*CsACO5*	Ortholog (Interspecific)	0.030696	0.325864	0.094200	Purifying Selection	10.86
*McACO5*_vs_*CmACO5*	*McACO5*	*CmACO5*	Ortholog (Interspecific)	0.029246	0.325687	0.089797	Purifying Selection	10.86
*AtACO4*_vs_*AtACO2*	*AtACO4*	*AtACO2*	Paralog (Intraspecific)	0.078369	0.864518	0.090651	Purifying Selection	28.82
*AtACO5*_vs_*CsACO5*	*AtACO5*	*CsACO5*	Ortholog (Interspecific)	0.156297	2.140470	0.073020	Purifying Selection	71.35
*AtACO2*_vs_*CmACO3*	*AtACO2*	*CmACO3*	Ortholog (Interspecific)	0.192588	3.032280	0.063513	Purifying Selection	101.08
*AtACO4*_vs_*CmACO3*	*AtACO4*	*CmACO3*	Ortholog (Interspecific)	0.188852	1.534470	0.123073	Purifying Selection	51.15
*AtACO1*_vs_*CmACO1*	*AtACO1*	*CmACO1*	Ortholog (Interspecific)	0.266962	4.000850	0.066726	Purifying Selection	133.36

Table S4. Expression stability of reference genes

Methods	Stability ranking（From left to right, stability gradually decreases）				
Delta CT	*HMG1/2*	*GAPDH*	*ACT7*	*PHOS32*	*TIP41*
BestKeeper	*HMG1/2*	*ACT7*	*GAPDH*	*PHOS32*	*TIP41*
Normfinder	*GAPDH*	*HMG1/2*	*ACT7*	*PHOS32*	*TIP41*
Genorm	*GAPDH* | *HMG1/2*		*ACT7*	*PHOS32*	*TIP41*
**RefFinder**	***HMG1/2***	***GAPDH***	***ACT7***	***PHOS32***	***TIP41***

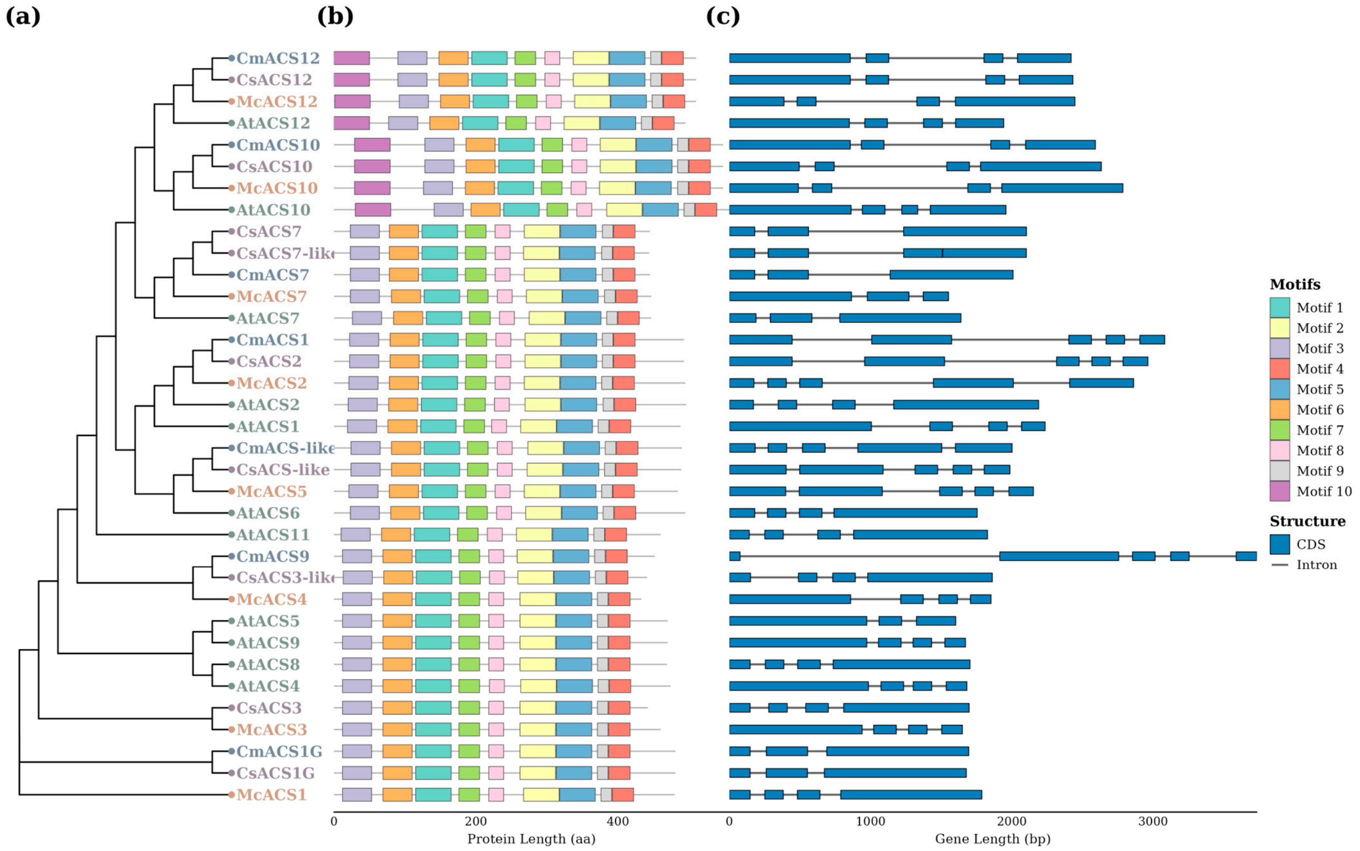

Figure S1. Phylogenetic relationships, conserved protein motifs, and gene structures of the *ACS* family across four representative species. (a) Maximum likelihood (ML) phylogenetic tree of 35 ACS proteins from *Momordica charantia* (Mc), *Arabidopsis thaliana* (At), *Cucumis sativus* (Cs), and *Cucumis melo* (Cm). (b) Distribution of conserved motifs identified using the MEME suite. Different motifs are represented by distinct colored boxes. (c) Exon–intron architectures of the corresponding *ACS* genes. The lengths of exons and introns are shown proportionally.

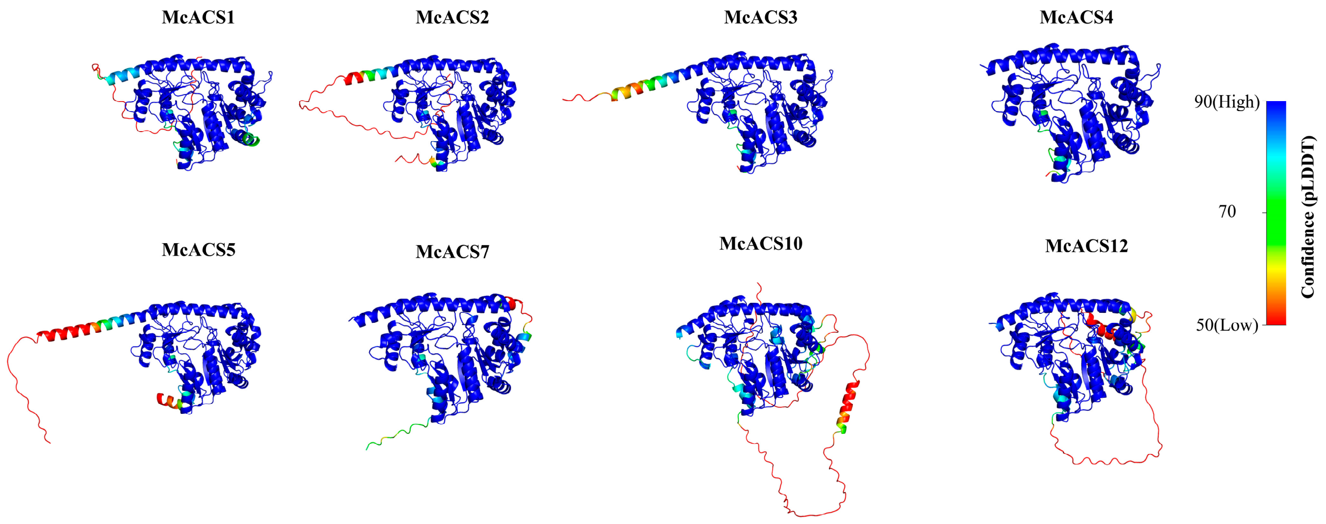

Figure S2. AlphaFold 3-predicted three-dimensional structures of all eight McACS proteins. The models are colored according to their pLDDT confidence scores. This visual mapping clearly highlights the diverse, intrinsically disordered C-terminal extensions (indicated by red/orange low-confidence regions) in contrast to the highly conserved and structurally rigid catalytic cores (indicated by deep blue high-confidence regions).

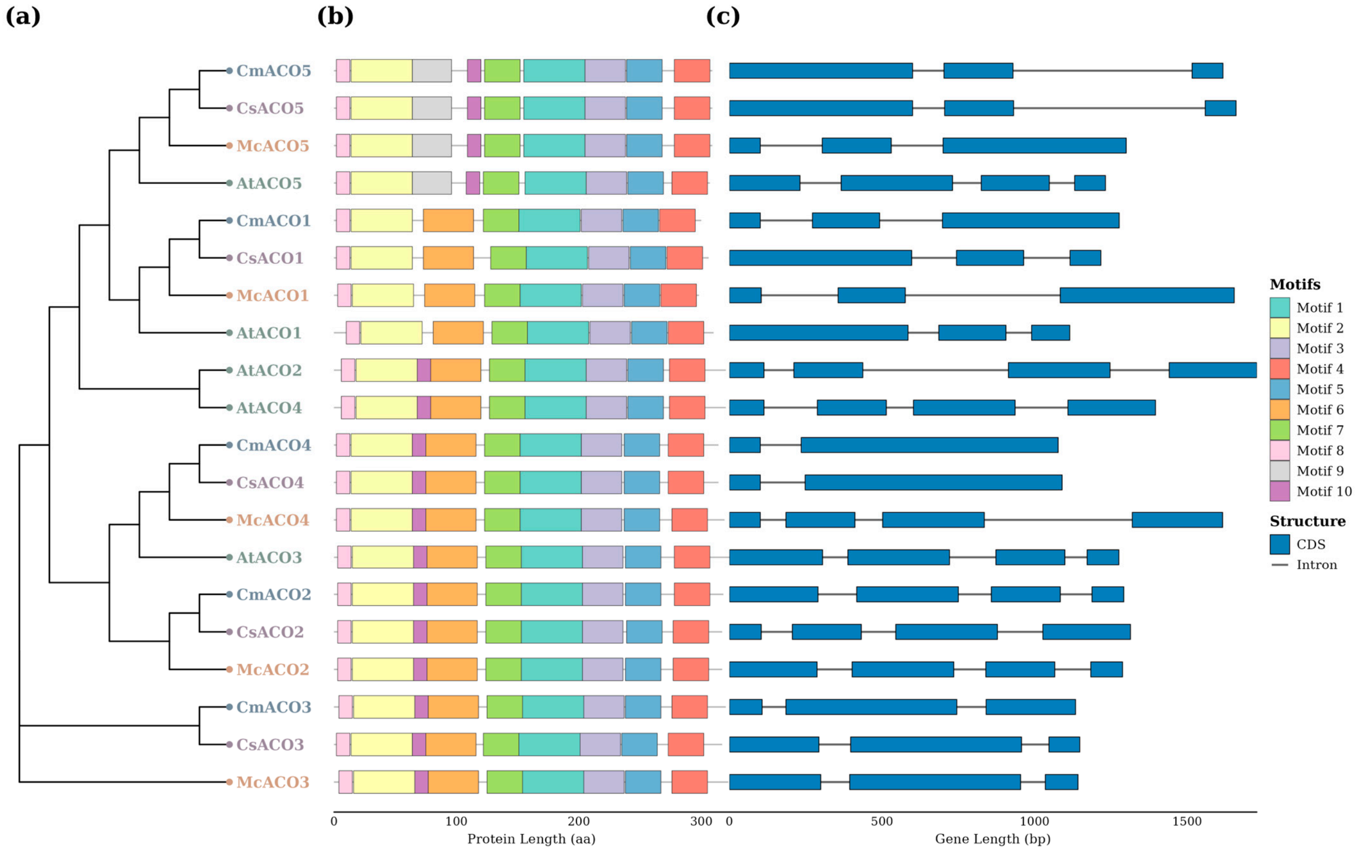

Figure S3. Phylogenetic relationships, conserved protein motifs, and gene structures of the *ACO* family across four representative species. (a) Maximum likelihood (ML) phylogenetic tree of 20 ACO proteins from *M. charantia*, *A. thaliana*, *C. sativus*, and *C. melo*. **(b)** Conserved protein motif compositions. **(c)** Exon–intron architectures of the *ACO* genes. Note the conservation of exon numbers in bitter gourd *McACOs* (three to four exons) compared to the lineage-specific exon fusion (two elongated exons) observed in *Cucumis* orthologs.

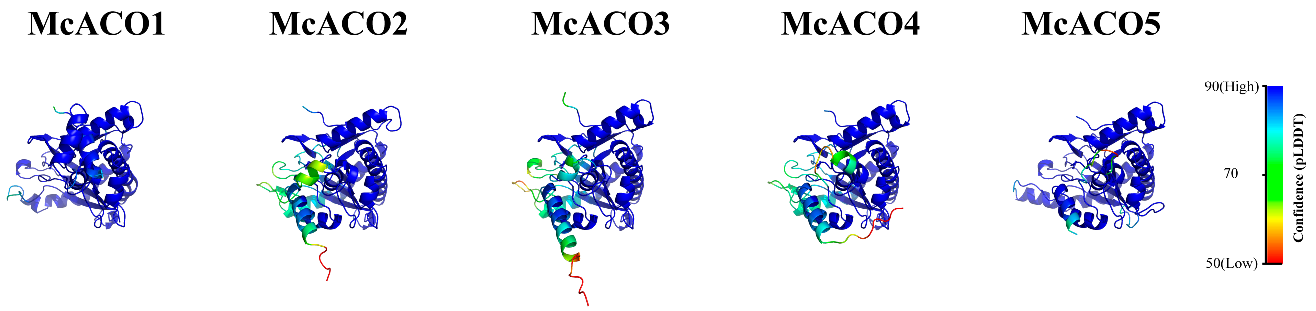

Figure S4. AlphaFold 3-predicted three-dimensional structures of the five McACO proteins. The high pLDDT scores (blue) across all members indicate a highly conserved and rigid jelly-roll β-barrel catalytic core, lacking flexible extensions.
